# Impact of Green Extraction on Curcuminoid Content, Antioxidant Activities and Anti-Cancer Efficiency (In Vitro) from Turmeric Rhizomes (*Curcuma longa* L.)

**DOI:** 10.3390/foods11223633

**Published:** 2022-11-14

**Authors:** Kanjana Singh, Somdet Srichairatanakool, Teera Chewonarin, Adchara Prommaban, Rajnibhas Sukeaw Samakradhamrongthai, Margaret Anne Brennan, Charles Stephen Brennan, Niramon Utama-ang

**Affiliations:** 1 Division of Product Development Technology, Faculty of Agro-Industry, Chiang Mai University, Chiang Mai 50100, Thailand; 2 School of Science, RMIT University, Melbourne 3000, Australia; 3 Cluster of High Value Products from Thai Rice and Plant for Health, Chiang Mai University, Chiang Mai 50100, Thailand; 4 Cluster of Innovative Food and Agro-Industry, Chiang Mai University, Chiang Mai 50100, Thailand; 5 Department of Biochemistry, Faculty of Medicine, Chiang Mai University, Chiang Mai 50100, Thailand; 6 Department of Wine, Food and Molecular Biosciences, Faculty of Agriculture and Life Science, Lincoln University, Christchurch 7647, New Zealand

**Keywords:** curcuminoid, turmeric, microwave-assisted extraction, ultrasound-assisted extraction, cytotoxic

## Abstract

Turmeric (*Curcuma longa* L.) powder is widely used as a spice and seasoning in Asian countries. This study investigated the effect of turmeric extracts on the anticancer activity of Huh7 and HCT 116 cells. The curcumin bioactive compounds were extracted using various methods such as microwave-assisted extraction (MAE), ultrasound-assisted extraction (UAE) and traditional extraction (TDE). The yield of dried extracts from MAE was found to be the highest at 17.89%, followed by UAE and TDE, with 11.34% and 5.54%, respectively. Antioxidant activities such as TPC, DPPH and FRAP from MAE were higher than those of UAE and TDE. The total curcuminoid contents from the novel extractions were higher than those from traditional extraction methods. For instance, curcuminoid contents from MAE, UAE and TDE were 326.79, 241.17 and 215.83 mg/g, respectively. Due to having the highest bioactive compounds and extraction yield, turmeric extract from MAE was used to investigate the potential anticancer properties. The extract showed significant cytotoxic potential against the human liver (Huh7) and human colon (HCT116) cell lines, in concentrations ranging from 31.25 to 1000.00 µg/mL. Turmeric extracts using MAE have potential anticancer effects on Huh7 and HCT116 cells. This study serves as scientific data for the chemotherapeutic properties of turmeric extracts and their use as functional ingredients.

## 1. Introduction

**Figure 1:**
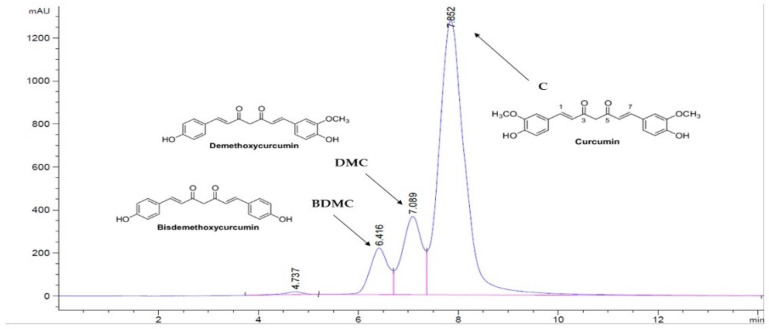
Chromatogram of turmeric extracts using HPLC and chemical structure of curcumin.

Phytochemicals from dietary sources have shown potential uses as therapeutic and chemo preventive agents for several chronic maladies [1]. Dietary phytochemicals of spices, and phenolic components of fruits and vegetables, have been shown to have significant potential in their ability for potent antioxidant activity, and suppression of carcinogenesis and other diseases in pre-clinical models [2,3,4]. Turmeric (*Curcuma longa* L.) is a rhizomatous herbaceous plant of the ginger family, Zingiberaceae, and has been traditionally used as an antiseptic for wound healing and as an anti-inflammatory agent due to its bioactive compounds and antioxidant activities [5]. Studies have shown that turmeric extracts have potential anti-viral and antitumor activities [6], and may therefore be useful for the prevention and treatment of such diseases. The compounds of turmeric extracts that possess biological properties are known as curcuminoids [7,8]. The typical curcuminoids include curcumin, demethoxycurcumin (DMC) and bis-demethoxycurcumin (BDMC) (Figure 1). Confirming this, Ahmad et al. [9] demonstrated that apoptosis and attenuation of telomerase activity in Huh7 and HCT116 cells were factors which accounted for the anti-proliferative effects of curcumin.

Different methods have been reported for the isolation of curcumin and other curcuminoids from turmeric powder, such as conventional solvent extraction, hot and cold percolation, and Soxhlet extraction and advanced or novel methods based on microwave-assisted extraction (MAE) and ultrasonic-assisted extraction (UAE) [10]. Conventional extraction methods are routinely associated and use high temperatures and large amounts of solvent, and have long extraction times and sometimes poor extraction efficiency [11]. Recently, research has evaluated cleaner extraction methods to reduce the environmental impact of such processes whilst still producing high-quality herbal extracts [12,13,14]. The electromagnetic energy of microwave extraction techniques can be converted to heat depending on the polarity of the solvent. MAE provides several interesting advantages, including high extraction yield, faster heating, reduced thermal gradients, and shorter reaction and preparation time [15]. In the thermal mechanism of UAE, the absorbed acoustic energy is turned to heat. The acoustic streaming leads to cavitation when passing through liquid and liquid-containing solid materials. UAE can save energy and time, as well as reducing extraction temperatures and the amount of solvent [16].

In Asian countries, turmeric is consumed as a seasoning and medical food. The markets are generally supplied with turmeric as fresh rhizomes and as a dried powder [17]. A detailed review of the literature reveals that there are few studies on curcumin extracts comparing both traditional extraction and novel extractions [18], no available reports discussing a comparison of the effects of MAE, UAE and TDE on turmeric, and also no quantified studies on curcuminoids using high-performance liquid chromatography (HPLC). Thus, this study aims to use traditional and novel extraction methods to extract the bioactive compounds, curcuminoids, for which turmeric extracts are usually consumed, from turmeric powder, and to compare the extraction efficiency of the different methods using the obtained extracts. In this study, MAE and UAE were used to extract curcuminoids and the samples were compared with those obtained using Soxhlet extraction, which was used as the reference method. Turmeric extracts from these methods were examined for their potential for cancer prevention in human liver cancer cell lines, and the potential development of a functional food and its application in the food industry.

## 2. Materials and Methods

### 2.1. Materials

Dried rhizomes of turmeric were purchased from Premium Foods Co., Ltd., Chiang Mai province, Thailand. Turmeric was ground by hammer mill machine with a 1.0 mm sieve and kept in aluminum foilbags under vacuum packing. Standard curcumin was purchased from Merck, Germany. Methanol, acetic acid, acetonitrile and water (HPCL grade) were obtained from Merck for the analysis and quantification of curcumin.

### 2.2. Extraction Methods

The traditional extraction technique (TDE) was carried out using the Soxhlet extraction. The turmeric power was attained by using 5 g of turmeric powder placed in Soxhlet apparatus, and subjected to extraction using 95% *v/v* ethanol at 60 °C for 8 h. The ethanol was separated from the extracts using a rotary evaporator under vacuum at 40 °C for 1 h (Buchi rotavapor R-200, Flawil, Switzerland).

UAE (Model VC505, Sonics & Materials, Inc., Newtown, CT, USA) was used for the extraction of turmeric powder using an ultrasonic power of 250 W and a frequency of 22 kHz [18]. For the extraction experiment, 5 g of turmeric powder was dissolved in 100 mL of ethanol and extracted in the ultrasonic machine for 3 h [11]. The ethanol was separated from the extracts using a rotary evaporator under vacuum at 40 °C for 1 h.

MAE (TOSHIBA, Model ER-300C (S)) was used for the extraction of turmeric powder by dissolving 5 g of turmeric powder in 100 mL of 95% *v/v* of ethanol and placing it in the microwave chamber. The turmeric was extracted at 800 W for 3 min. The extraction was performed in cycles with 30 s of irradiation and 5 min of cooling time to control a temperature of 25 °C and to avoid solvent boiling [19].

### 2.3. Physical and Chemical Analysis and Yield of Extraction

AOAC methods were used for the determination of proximate analysis such as protein, fat, ash and moisture content [20]. A Konica Minolta colorimeter (CR-400 Series, Tokyo, Japan) was used for color determination of the powder and an AquaLab Water Activity Meter (Decagon, Device, Inc, NE Hopkins Ct, Pullman, WA, USA) determined water activity. The *Yield* of dried extracts, on a dry weight basis, was calculated from Equation (1) shown below:

(1) $$Yield\;\left(\%\right)=\frac{w1\times 100}{w2}$$

where *w*1 was the weight of extract after evaporation of ethanol and *w*2 was the dry weight of turmeric powder.

### 2.4. Antioxidant Activities: Total Phenolic Content (TPC), DPPH and FRAP

A 200 μL sample was mixed with 1.0 mL of Folin-Ciocaltue’s reagent and 0.8 mL of Na_2_CO_3_ 7.5%. Sample absorbance values were measured at 765 nm, after incubating at room temperature for 30 min, and results were expressed as milligrams of gallic acid equivalent (GAE) per gram of dry material [21].

One millilitre of DPPH radical solution (0.1 mM in methanol) was mixed with 3 mL of methanolic solution of the extracts. The reaction mixture was agitated with a vortex mixer and allowed to stand for 30 min in the dark at room temperature. The absorbance was recorded at 517 nm using a spectrophotometer [22].

FRAP analysis was conducted according to the method of Singh et al. [22]. In this step, 200 μL of extract solution was mixed with 1.5 mL of the FRAP reagent, and incubated at 37 °C for 4 min. The absorbance of the solution was measured at 593 nm. A standard curve was plotted using an aqueous solution of ferrous sulphate (FeSO_4_⋅7H_2_O) (100–1000 μmol), with FRAP values expressed as micromoles of ferrous equivalent (μM Fe [II]) per 100 g of sample.

### 2.5. Analysis of Curcuminoid Content Using High Pressure Liquid Chromatograph (HPLC)

The turmeric extracts (0.0100 g) were mixed with 10 mL of ethanol and diluted to obtain a 250 µg/mL concentration. A standard solution of curcuminoid was prepared by mixing curcumin (80%), DMC (17%) and BDMC (3%) (Sigma, St. Louis, MI, USA). Reversed-phase HPLC analysis was conducted on an Agilent HPLC system (1200 series, Boeblingen, Germany), consisting of a binary pump and a diode-array detector (DAD) and equipped with 250 × 4.6 mm i.d., 5 µm Restek C18 column (Restek, Bellefonte, PA, USA). The mobile phase consisted of 1% (*v/v*) acetic acid in filtered MilliQ water (solvent A) and acetonitrile (solvent B) using the following profile: 0–20 min, 40% solvent B; 20–32 min, 60% solvent B; 32–38 min, 100% solvent B; and from 38–40 min re-equilibrated back to 40% solvent B with a flow rate of 1 mL/min (Singh et al., 2017). The signal was observed at 425 nm. Curcuminoid content was determined against standard curves (Figure 1) (linearity of R2 >0.98). The LODs of BDMC, DMC and C were 1.22, 0.28, 0.57 µg/mL, respectively, and the LOQs of BDMC, DMC and C were 3.72, 2.27, 1.74 µg/mL, respectively.

### 2.6. Human Cell Culture

Dulbecco’s Modified Eagle Medium (DMEM) with the addition of 10% fetal bovine serum, penicillin (100 IU/mL) and streptomycin (100 IU/mL) were used for the culture of Huh-7 and HCT-116 at 37 °C, following previously published methodology [19], and cells were harvested at 80–90% confluence.

### 2.7. Cell Viability Assay

Cell viability was assessed using 3-(4,5-dimethylthiazol-2-yl)-2,5-diphenyl-2H-tetrazolium bromide (MTT assay) [23]. In this step, Huh-7 cells and HCT-116 cell were treated with extracts (0–200 μg/mL, final ethanol concentration 0.4%) for 24 and 48 h, washed with PBS and then incubated with MTT (5 mg/mL) for 4 h. The resulting MTT formazan product was dissolved with DMSO (0.1 mL), and the OD was measured at 570 nm. The percentage of cell viability was plotted against extract concentration.

### 2.8. Statistical Analysis

The experimental data were subjected to statistical evaluation using analysis of variance (Two-Way ANOVA) for a completely random design. Duncan’s multiple range tests were used to determine the difference between means and the significance was defined at *p* < 0.05. The half-maximal inhibitory concentration (IC50) was analyzed by nonlinear regression using GraphPad Prism (version 8.0, GraphPad Software).

## 3. Results

### 3.1. Proximate Composition of Dried Turmeric

The turmeric powder color was analyzed using CIELAB. The L* was 42.33, which indicated the lightness. The a* was 10.43, which indicated redness and b* was 32.70, which presented a shift toward yellow. The water activity of the turmeric powder was 0.487, which conformed to the dried food specification (aw ≤ 0.6). The moisture content was 9.85% on the dry weight basis, which was concordant with the Thai industrial standard limit of 10%. Protein content, carbohydrate, crude fiber, lipid and ash were 6.43, 60.53, 9.33, 4.89 and 8.98, respectively. The total phenolic content was 41.93 mgGAE/g DW, and contents of antioxidants DPPH and FRAP were 17.77 mgGAE/g DW and 25.34 mg Fe (II)/g DW, respectively (Table 1). The content of curcuminoids, including curcumin, DMC and BDMC (Table 2) were analyzed using HPLC and were found to be 50.43, 26.03 and 11.76 mg/g dry weight, respectively.

### 3.2. Effect of Ultrasonic-Assisted Extraction (UAE)

Organic solvents have been used to extract turmeric powder by UAE (acetone, methanol and ethanol). Sahne et al. [24] found that the UAE of turmeric using 95% ethanol was attributed to favorable properties, such as high polarity, low viscosity, surface tension, absorption of ultrasound energy and high yield of extraction compared with other solvents. This is the reason why 95% *v/v* of ethanol was used in the UAE for this study. Table 1 illustrates an 11.34 % extraction yield of turmeric extract from UAE. Research has illustrated that the polarity of ethanol increases the permeability of the cell wall and improves extraction yield [25]. The ethanol solvent determines the cavitation intensity based on the phytochemical properties and also had a high capacity to absorb and transmit the energy of the ultrasound during UAE.

In this study, an ultrasonic power of 250 W and ultrasound frequency of 22 kHz were selected from a previous study which evaluated the optimum ultrasonic power and frequency for turmeric extraction [24]. Table 1 illustrates that the total phenolic content (TPC) of the extract was 112.5 mg GAE/g, and the antioxidant properties of DPPH and FRAP were 64.32 mg GAE/g and 77.82 mg FE (II)/g, respectively. The content of curcuminoids, including curcumin, DMC and BDMC were analyzed using HPLC. The contents of curcumin, DMC and BDMC were 117.44, 83.54 and 40.19 mg/g dry extracts, respectively (Table 2). Previous research has illustrated that ultrasonic power and frequency also affects the turmeric extracts [26].

Ultrasonic power applied to the larger amplitudes of ultrasound can increase the collapse of cavities or damaged cell walls, which in turn can increase the solute diffusion interfacial turbulence and local energy dissipation [27]. Degradation of extracted compounds was also observed when using too high an ultrasonic power. Thus, an optimized ultrasonic power must be identified before extraction [28]. The frequency of ultrasonic power aids cavitation bubbles and in turn can create micro-turbulence and inter-particle collisions in the micro-porous particles of the plant material, resulting in the acceleration of diffusion. Swamy & Narayana [29] reported that the time taken in the distillation cycle for bubbles to grow to a large size in order to accumulate energy is shorter at high frequencies, thereby reducing the degree of cavitation intensity. In this study, the temperature of the solvent was controlled during extraction (40–45 °C). An increase in temperature may also interrupt and open the cell matrix, leading to increased amounts of curcumin in solution during extraction. However, lower recoveries of active ingredients also occur at significantly higher temperatures [30].

### 3.3. Effect of Microwave-Assisted Extraction (MAE)

Solvent type is one of the most important parameters for efficient MAE. For instance, the choice of solvent has to consider the affinity of the target compound and also the ability to absorb microwave energy. Many organic solvents have been used to extract turmeric powder, such as acetone, methanol, ethanol and propylene glycol [31]. The report by Ravindran et al. [6] showed that MAE using ethanol produced the highest curcuminoid content and yield of extraction, as well as a lower toxicity, followed by methanol and propylene glycol. Curcuminoid extracts obtained using MAE with 95% ethanol produced the highest yield of curcuminoid content, extraction yield and bioactive compounds, because 95% ethanol was found to have an optimum dielectric constant, viscosity and solubility for the target compound. For this study, 95% *v/v* ethanol was used in the MAE. From Table 1, it can be seen that the extraction yield of turmeric extract using MAE was 17.89% and the moisture content was 12.21%. MAE is the one of the techniques that has been perceived as an advantageous technique, improving the extraction of bioactive compounds and diffusion of solvent. The electromagnetic energy of microwaves is converted to heat. This conversion depends on the polarity of the utilized solvent. Because ethanol absorbs microwave energy efficiently, MAE using this solvent results in faster heating of compounds, reduced thermal gradients and high extraction yields compared with other solvents [15]. The study by Singh et al. [22] optimized the microwave power and time for turmeric extraction and found that 800 W of microwave power for 3 min provided a high efficiency in obtaining turmeric extracts and other bioactive compounds: these optimum conditions were therefore selected in this study. The content of TPC was 178.36 mg GAE/g extract, and those of antioxidants DPPH and FRAP were 72.54 mg GAE/g and 82.11 mg FE (II)/g, respectively (Table 1). The content of curcuminoids, including curcumin, DMC and BDMC (Table 2) were analyzed using HPLC and were found to be 186.84, 95.12 and 45.03 mg/g dry extracts, respectively. Microwave power and time influences the curcuminoid content and bioactive compounds of turmeric extracts, as reported by Singh et al. [22] and Laokuldilok et al. [31]. The higher temperature caused by the microwave power during microwave radiation could hydrolyze the ether linkages of the cellulose and convert them into soluble fractions within 1 to 2 min. After that, the higher temperature experienced by the cell walls during microwave radiation of cellulose could reduce microwave radiation and could also have enhanced the dehydration of cellulose and reduced its mechanical strength, which would enable easy access of the solvent into the compounds inside the cell. When the internal pressure increases beyond the stability of the cell wall, it ruptures the cellular structure of the turmeric powder. The breakup of the cell wall then removes the major resistance to mass transfer to and from the cell structure. This results in an increase in the mass transfer rates of active constituents during extraction. Thus, the curcuminoid content increases with increasing microwave exposure time. The research of Dandekar & Gaikar [32] reported that the optimum microwave power and time should be identified because the higher temperature and longer exposure time during microwave irradiation might increase the loss of curcumin due to the thermal degradation of active constitutions.

### 3.4. Effect of Traditional Extraction (TDE)

Soxhlet extraction was used to compare the efficiency of the methods for turmeric extraction, as a reference method. An important consideration is that the solvent should be easy to remove, and inert. Research has focused on selecting solvents based on the increasing polarity order, such as the order of acetone, petroleum ether, ethyl acetate, chloroform, methanol, ethanol and water [26]. For instance, Sahne et al. [24] reported that 95% ethanol is the best solvent for turmeric extraction using TDE. The study of TDE extraction of Chhouk et al. [33] was chosen, and 95% ethanol was used. As can be seen from Table 1, the extraction yield of turmeric extract using TAE was 5.54% and the moisture content was 11.55%. The disadvantage of this extraction method is that the solvent is constantly heated at its boiling point and the extraction takes a long time, which can damage thermo-labile compounds. The TPC of the extracts was 97.14 mg GAE/g, and the antioxidant properties of DPPH and FRAP were 60.07 mg GAE/g and 72.72 mg FE (II)/g, respectively (Table 1). The content of curcuminoids including curcumin, DMC and BDMC were analyzed by using HPLC, and the contents of C, DMC and BDMC were found to be 95.57, 81.70 and 38.56 mg/g dry extracts, respectively (Table 2).

### 3.5. Comparison of UAE, MAE and TDE

**Figure 2:**
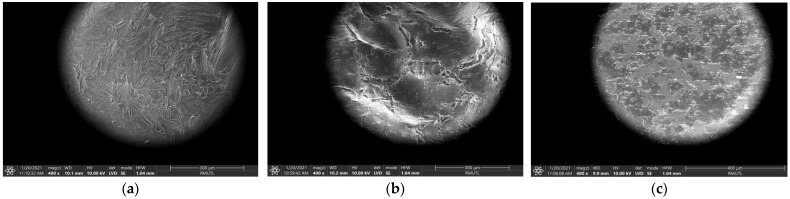
Scanning electron micrograph of turmeric extracts using UAE (**a**), MAE (**b**) and TDE (**c**).

Turmeric extraction using UAE and MAE, as novel extractions, was compared with a traditional assisted extraction as a reference method. The scanning electron micrograph has been shown in Figure 2. The UAE and MAE methods of extraction have been shown to reduce operating time, and achieve a higher level of bioactive yield [34]. The extraction times for curcuminoid extraction using UAE, MAE and TDE were 15 min, 3 min and 6 h, respectively. The curcuminoid extraction yields using UAE, MAE and TDE were significantly different (*p* < 0.05). Bioactive compounds such as TPC, antioxidant activities including those of DPPH and FRAP, and curcuminoid content including curcumin, DMC and BDMC are rarely compared using three different extraction methods. Table 1 and Table 2 show that the highest levels of bioactive compounds and curcuminoid content were found using MAE, followed by UAE and TDE (*p* < 0.05).

Mandal et al. [35] reported that the extraction rate of novel extraction methods including MAE and UAE were 27% more efficient compared with the conventional method, and used lower temperatures and shorter times. This showed that the traditional extraction method did not perform an effective extraction of turmeric compared with MAE and UAE. Sonication disrupts plant cell walls by inducing cavitation and allows solvent penetration to the plant matrix and enhancement of turmeric by UAE. Meanwhile, MAE causes direct rapid heating due to microwave-induced molecule dipole rotation and ionic conduction, which causes the cell to break and facilitates the release of curcuminoids into the extracting solvent. Thus, the lower extraction time and operating temperature are the major advantages of novel extraction compared with the traditional methods.

### 3.6. Anticancer Activity from Turmeric Extracts on Huh7 Cells and HCT116 Cells Using Cell Viability Assay

As already reported, curcuminoids have a strong anti-proliferative effect on cancer cells [1]. Therefore, the turmeric extracts obtained using MAE from a previous study were used to investigate the effect of curcumin standard and turmeric extracts on Huh7 and HCT116 (cancer cell) cell viability at increasing concentrations (0, 31.25, 62.50, 125.00, 250.00, 500.00 and 1000.00 µg/mL after 24 h and 48 h of treatment.

**Figure 3:**
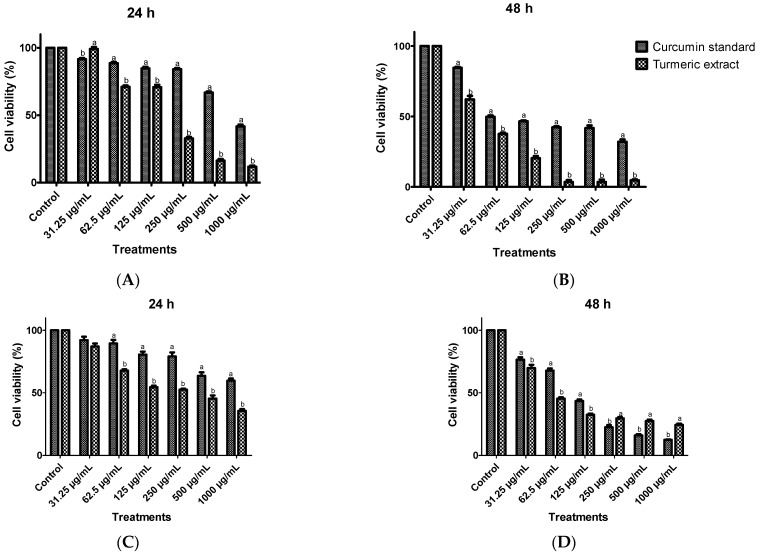
The viability of Huh7 (**A**,**B**) and HCT116 (**C**,**D**) cancer cells treated with standard curcumin and turmeric extracts at the indicated concentrations for 24 h and 48 h were determined using an MTT assay ^a–b^ (*p* < 0.001).

**Figure 4:**
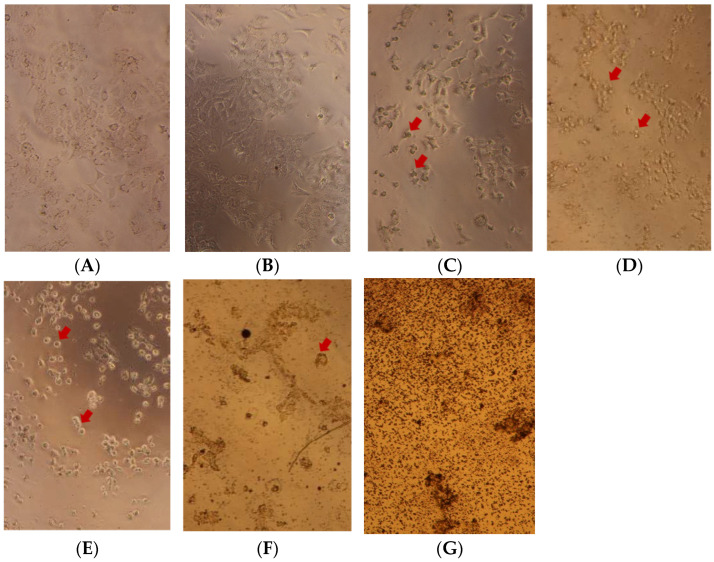
Direct microscope observations of Huh7 cancer cells treated with turmeric extracts at different concentrations ((**A**); 0, (**B**); 31.25, (**C**); 62.50, (**D**); 125.00, (**E**); 250.00, (**F**); 500.00 and (**G**); 1000.00 µg/mL) under a microscope with 10× magnification compared with untreated control (**A**); red arrows present the morphology change of death cells.

As reported in Figure 3 and Figure 4, a significant time- and dose-dependent decrease in cell viability was observed in both cell lines. With 31.25 µg/mL of turmeric extract, there was a slightly decreased cell viability of Huh7 treated cells at 24 h and 48 h, reaching about 99.24% and 62.39% whereas curcumin standard at 24 h and 48 h reached at 91.18% and 84.37%, respectively. Similar to HCT116 cells, at 31.25 µg/mL of turmeric extract, the cell viability of treated cells at 24 h and 48 h reached about 87.06 and 69.76 whereas curcumin standard reached 92.04 and 76.41, respectively. The half-maximal inhibitory concentration (IC50) of turmeric extracts and curcumin standard on Huh7 treated cells at 24 h were 159.6 and 832.07 µg/mL, respectively, whereas at 48 h these were 43.28, 41.22 µg/mL, respectively. The treated HCT116 cells showed a similar trend to the Huh7 cells, in which the IC50 of turmeric extracts at 24 h was 66.37 µg/mL whereas the IC50 concentration of curcuminoid standard was more than 1000.0 µg/mL. The IC50 of cells treated with turmeric extracts and curcumin standard at 48 h were 37.27 and 88.16 µg/mL, respectively. An increase in the concentration of turmeric extracts and curcumin standard significantly decreased cell viability; turmeric extracts significantly decreased cell viability compared with curcumin standard. It could be indicated that turmeric extracts are enriched with phenolic compounds as well as various curcuminoid compounds, which reveals potent antioxidant activity and induces anti-cancer activity. Several previous studies demonstrated that turmeric extract had anti-cancer activity, supporting our investigation. Previous literature found that turmeric extract possessed anti-cancer activity which was carried out through direct scavenging of oxygen radicals and stimulation of antioxidant responses by nuclear factor erythroid 2-related factor 2 (Nrf2) activation [9]. Moreover, the anti-proliferative effects of curcumin were accompanied by pronounced apoptosis and attenuation of telomerase activity in Huh7 liver cancer cells [36].

Chuang et al. [37] found that curcumin treatment 0.2% reduced carcinoma cells in male mice by reducing p21 protein, thereby protecting against genome destabilization that occurs before entering the cancer state. The study of curcumin absorption in the human body found that curcuminoid extract from turmeric rhizome is absorbed to a greater extent in the human body than pure curcumin [38]. Turmeric extract contains a complex of curcumin with phospholipids and volatile oil which increases the relative human absorption. As we know, turmeric and curcuminoid are commonly used as food, seasoning and medicine. Our findings indicated that turmeric extract has the potential to reduce both Huh7 and HCT116 cell viability and could be applied as a supplementary food at a suitable dose. Furthermore, turmeric extract is suggested for intense anti-cancer investigation to support anti-cancer therapy in the future.

## 4. Conclusions

In the present work, an eco-friendly extraction method using MAE and UAE was shown to be an effective alternative to traditional extraction methods for the isolation of curcuminoids from turmeric powder. The study has confirmed that MAE and UAE increase the extraction rates compared with TDE. The obtained results demonstrated the lower extraction time of MAE, which is performed at lower temperatures compared with conventional methods. Overall, MAE showed significant potential to improve curcumin extraction, and reduce the extraction cost; it uses an eco-friendly system due to the lower demand for heating, and takes a shorter time and has a higher extraction yield when compared with conventional extraction systems. The MAE extracts also possessed good anticancer activities. Promising in vitro anticancer activity against Huh7 and HCT116 cell lines was observed at a range of 6.25 to 200.00 µg/mL. Defining the adequate concentration of curcuminoids and optimal processing and storage conditions can be seen as the next challenges for researchers for further development in the food industry.

## Figures and Tables

**Table 1:** Extraction yield, total phenolic content and antioxidant properties: total phenolic content, DPPH and FRAP of turmeric powder and turmeric powder obtained by different extraction techniques ^1^.

Extraction Technique	Extraction Yield (%, DW)	TPC (mgGAE/g DW)	DPPH (mgGAE/g DW)	FRAP (mg Fe (II)/g DW)
Turmeric powder	-	41.93 ± 4.28 ^d^	17.77 ± 3.90 ^d^	25.34 ± 5.43 ^d^
MAE	17.89 ± 1.43 ^a^	178.36 ± 4.76 ^a^	72.54 ± 4.70 ^a^	82.11 ± 6.09 ^a^
UAE	11.34 ± 2.48 ^b^	112.50 ± 8.46 ^b^	64.32 ± 3.64 ^b^	77.82 ± 4.58 ^b^
TDE	5.54 ± 0.98 ^c^	97.14 ± 10.04 ^c^	60.07 ± 4.87 ^b^	72.72 ± 5.45 ^b^

^1^ Values are the mean ± standard deviation (*n* = 3); DW: dry weight; MAE: microwave-assisted extraction; UAE: ultrasonic-assisted extraction; TDE: traditional extraction; ^a–d^ represents significance difference in the columns as *p* < 0.05.

**Table 2:** Curcuminoid content of turmeric powder and turmeric powder extracted by different extraction techniques ^1^.

Extraction Technique	C (mg/g DW)	DMC (mg/g DW)	BDMC (mg/g DW)
Turmeric powder	50.43 ± 5.51 ^d^	26.03 ± 1.56 ^d^	11.76 ± 2.88 ^d^
MAE	186.64 ± 9.73 ^a^	95.12 ± 1.45 ^a^	45.03 ± 1.02 ^a^
UAE	117.44 ± 5.94 ^b^	83.54 ± 3.64 ^b^	40.19 ± 8.73 ^b^
TDE	95.57 ± 6.65 ^c^	81.70 ± 2.40 ^b^	38.56 ± 6.91 ^b^

^1^ Values are the mean ± standard deviation (*n* = 3); DW: dry weight; C: curcumin; DMC: Demethoxycurcumin; BDMC: Bisdemethoxycurcumin; MAE: microwave-assisted extraction; UAE: ultrasonic-assisted extraction; TDE: traditional extraction; ^a–d^ represents significance difference in the columns as *p* < 0.05.

## Data Availability

The data generated during the current study are available from the corresponding author on reasonable request.
